# Training certified detectives to track down the intrinsic shortcuts in COVID-19 chest x-ray data sets

**DOI:** 10.21203/rs.3.rs-2818347/v1

**Published:** 2023-04-28

**Authors:** Ran Zhang, Dalton Griner, John W. Garrett, Zhihua Qi, Guang-Hong Chen

**Affiliations:** University of Wisconsin–Madison; University of Wisconsin–Madison; University of Wisconsin–Madison; Henry Ford Health; University of Wisconsin–Madison

## Abstract

Deep learning faces a significant challenge wherein the trained models often underperform when used with external test data sets. This issue has been attributed to spurious correlations between irrelevant features in the input data and corresponding labels. This study uses the classification of COVID-19 from chest x-ray radiographs as an example to demonstrate that the image contrast and sharpness, which are characteristics of a chest radiograph dependent on data acquisition systems and imaging parameters, can be intrinsic shortcuts that impair the model’s generalizability. The study proposes training certified shortcut detective models that meet a set of qualification criteria which can then identify these intrinsic shortcuts in a curated data set.

## Introduction

Deep learning has been incredibly successful in object detection, image classification, and natural language processing over the past ten years due to its ability to learn complex features from data. However, despite its success on benchmark datasets, there are limitations and practical issues when using these models in real-world scenarios. A major challenge is poor generalizability, where performance significantly drops when applied to external datasets^1–3^. This limits the translation and deployment of deep learning models for high-stakes tasks, such as in healthcare applications. This lesson was evident during the COVID-19 pandemic, where many machine learning models were developed, but very few performed well on real-world clinical tests^4–6^.

The concept of shortcut learning has recently been explored in deep learning studies^7^. It has been discovered that poor model generalizability can be attributed to shortcut learning when the training dataset has hidden shortcuts, meaning there are spurious correlations between irrelevant image features and the corresponding training labels. This causes models to quickly pick up these spurious correlations instead of the desired image features, establishing incorrect connections between input image data and output labels. For instance, early studies have shown that deep learning models can differentiate chest x-rays from different hospitals and patient groups^1^. This suggests that different data sources and patient characteristics like gender, age, and race could also become shortcuts.

**Figure 1:**
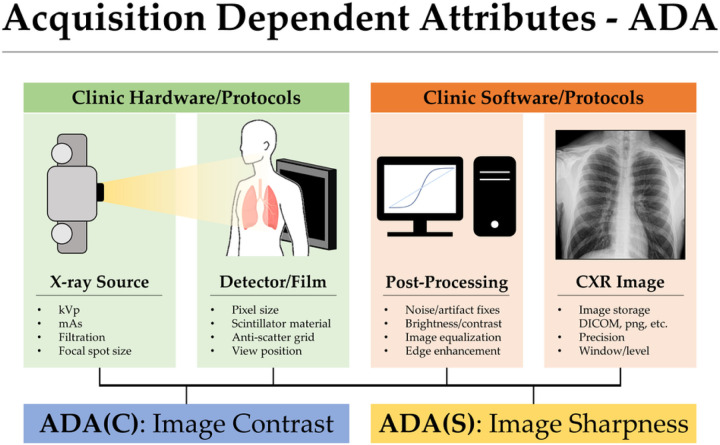
Acquisition Dependent Attributes (ADA) in chest x-ray images.

To illustrate the issue of shortcut learning in real-world clinical scenarios, let’s take the example of COVID-19 classification using chest x-ray radiographs (CXRs). DeGrave et al. ^8^ discovered that if COVID-19 positive and negative training data were collected from two different sources, the model would only learn the source label as a shortcut for prediction. As a result, the model would not have the desired prediction power in real-world clinical scenarios. The authors also found that the trained model used extrinsic image features, such as lead markers, for prediction, even though these markers only indicated the orientation of patients in x-ray image acquisitions and did not correspond to any disease features. Some suggestions have been made to remove these extrinsic shortcuts by a segmentation step^9^. However, even with these markers removed from the training dataset, some other shortcuts still existed in the segmented lung tissue-only training dataset^10^. As a result, the nearly perfect performance of the trained model^9^ is still not generalizable to real-world clinical datasets. The remaining shortcuts may be attributed to the inherent defining features of CXRs, such as image contrast and sharpness, which can vary from hospital to hospital due to different types of imaging systems, generations of x-ray imaging equipment, hardware components, image post-processing methods used by vendors, and imaging protocols used by technologists. All these factors can impact the digital representation of the acquired image data in terms of variations in image contrast and sharpness. Figure 1 demonstrates these variations.

Unlike other shortcuts that have been previously studied, such as age, gender, race, and markers, which are extrinsic and can be removed through careful data collection and cleaning, contrast- and sharpness-related shortcuts are more difficult to detect and mitigate. This is because desired image features are also represented as image contrast and spatial correlations, which are similar to the features of contrast- and sharpness-related shortcuts. This entanglement between desired image features and shortcut features makes studying contrast and sharpness-related shortcuts particularly challenging. Consequently, these shortcuts are referred to as intrinsic shortcuts in this work.

In order to develop an effective strategy for mitigating contrast- and sharpness-related shortcuts, it is necessary to first develop reliable methods for detecting their presence and severity within a carefully curated dataset. While post-hoc model interpretability methods, such as class activation maps^11^ and expected gradient^12^, have been developed to identify relevant image features used by trained deep learning models for prediction, these methods are unable to detect intrinsic shortcuts within a curated training dataset prior to model training. Furthermore, studies have suggested that these methods may not be effective in diagnosing poor generalization performance of the model^13^.

In this paper, we present a novel approach for detecting contrast- and sharpness-related intrinsic shortcuts using certified shortcut detective models. Our approach involves establishing qualification standards for suspected intrinsic shortcuts, designing a training curriculum for training the shortcut detectives to detect these shortcuts, performing certification tests on the trained detectives, and finally deploying them to curated datasets to examine the suspected shortcuts. We applied this approach to the available COVID-19 datasets to assess their quality. Our results demonstrate the effectiveness of this approach in detecting and mitigating intrinsic shortcuts.

## Methods

### Datasets

**Figure 2:**
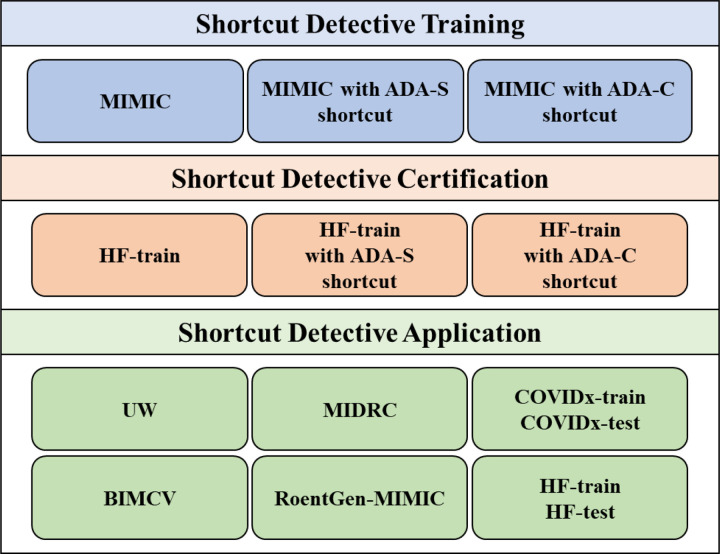
An overview of the datasets used in this work.

Figure 2 provides an overview of the datasets utilized in this study. The MIMIC-CXR dataset served as the training data for the shortcut detectives, while the HF-train dataset, a privately curated COVID-19 chest x-ray dataset, was utilized for certification tests of the trained detectives. The trained shortcut detectives were then applied to a variety of public and private chest x-ray datasets. Specific information about each dataset is provided below.

### MIMIC dataset

MIMIC-CXR^14^ chest x-ray dataset consists of 377,110 CXRs from 65,379 patients presenting to the Beth Israel Deaconess Medical Center Emergency Department between 2011–2016. In this work, 46,894 frontal-view (AP/PA) normal CXRs (cases with “No Finding” labels) were used to train shortcut detectives.

### HF dataset

This is a privately curated COVID-19 CXR dataset from patients presenting to the Henry Ford Health between March 1, 2020, and October 31, 2020. The COVID-19-positive and COVID-19-negative cohorts are collected within the same time range, from the same hospitals, and labeled by their most recent RT-PCR test result seven days before or after the imaging study. For model training and internal testing, two data partitions are generated: **HF-train** consisted of 8,733 COVID-19-positive CXRs from 4,383 patients and 16,584 COVID-19-negative CXRs from 8,733 patients; **HF-test** consisted of 695 COVID-19-positive CXRs from 526 patients and 8,878 COVID-19-negative CXRs from 6,081 patients.

### BIMCV dataset

This is a public COVID-19 CXR dataset collected in Spain^15^. This dataset was collected from 11 hospitals in the Valencian Region, Spain, between February and April 2020. After data curation, the dataset consisted of 4,169 COVID-19-positive CXRs from 2,663 patients and 5,050 COVID-19-negative CXRs from 3,710 patients.

### UW dataset

This is a privately curated COVID-19 CXR dataset. It includes consecutive patient cases from the University of Wisconsin Hospitals and Clinics (UW Health) from March 2020 to September 2021. The dataset comprised 1,025 COVID-19-positive CXRs from 658 patients and 8,774 COVID-19-negative CXRs from 5,953 patients.

### MIDRC dataset

This is a large, multi-institution public COVID-19 CXR dataset curated and released by the Medical Imaging & Data Resource Center (MIDRC). A total of 6,453 COVID-19-positive CXRs from 5,199 patients and 20,072 COVID-19-negative CXRs from 9,947 patients were pulled from the MIDRC Data Commons (https://data.midrc.org/, date accessed: December 7th, 2022).

### COVIDx dataset

This is a public COVID-19 CXR dataset released by the COVID-Net Open Source Initiative^16^. A total of 29,986 CXRs from 16,648 patients are included in the training dataset (**COVIDx-train**), and 400 CXRs are included in the test dataset (COVIDx-test). (Data downloaded from https://www.kaggle.com/datasets/andyczhao/covidx-cxr2?select=competition_test, date accessed: December 7th, 2022).

### RoentGen-MIMIC dataset

This dataset contains 943 synthetic CXRs generated by the RoentGen model^17^ and 1,000 real “No Finding” CXRs from the MIMIC dataset. The RoentGen model, which is trained using the MIMIC dataset, is able to generate visually convincing synthetic CXRs with different pathologies. To generate the synthetic CXRs used in this work, a text prompt of “No finding” was used as the input, only frontal view CXRs are included.

### Training and certification of shortcut detectives

#### Overview of the proposed framework

**Figure 3:**
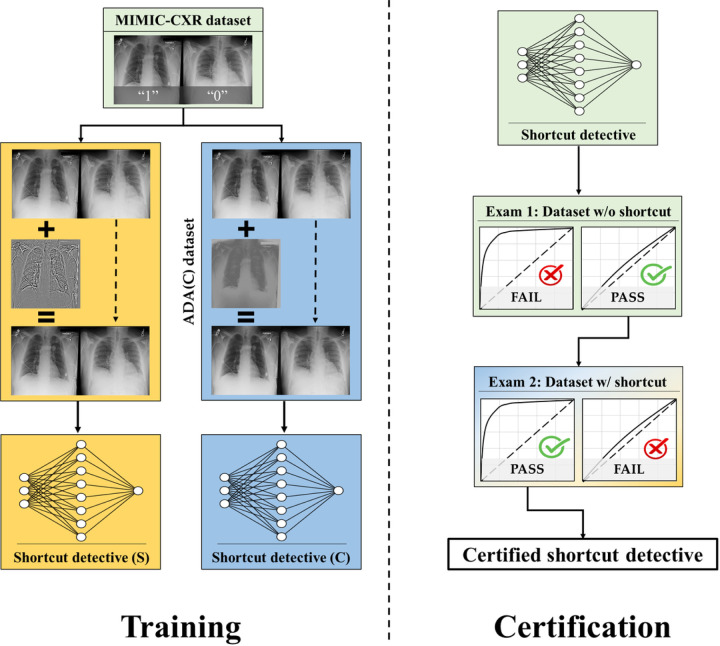
A framework to train and certify shortcut detectives.

An overview of the shortcut detective training and certification process is shown in Fig. 3.

To begin, 46,894 normal CXRs from the MIMIC dataset were selected and randomly divided into two equal groups: 23,447 of the CXRs were assigned as the positive class (“1”) while the rest were assigned as the negative class (“0”).

Then, to construct the training dataset for the shortcut detective, the image contrast or the image sharpness of the positive class were adjusted using the approach shown in Appendix A1. Since only normal CXRs were included, there were no disease-specific features present that could be used to distinguish between the two classes. Therefore, if a model was able to differentiate between the two classes, it would be because the model had learned the corresponding global image contrast or sharpness characteristics, rather than disease features.

Subsequently, shortcut detectives (neural network models for binary classification) were trained using the constructed training datasets with contrast or sharpness shortcuts. Details of the training are discussed in the following section and in Appendix A2.

To assess the efficacy of the shortcut detectives on COVID-19 CXR dataset, two types of examinations are necessary. Firstly, when the shortcut detective is deployed on a COVID-CXR dataset without the corresponding shortcut, it should not be able to differentiate between the COVID-positive and COVID-negative classes. Hence, an Area Under the Receiver Operating Characteristics curve (AUC) close to 0.5 is expected. This examination is crucial to ensure that the image features utilized by the shortcut detectives are not interwoven with the original imaging task. Secondly, when the shortcut detective is applied to a COVID-CXR dataset with a known shortcut, it should demonstrate superior classification performance, ideally with an AUC close to 1. For the first examination, the HF-train dataset is utilized. As the positive and negative cohorts are gathered within the same timeframe and from the same hospitals, it is expected that no contrast and sharpness shortcut exists. This has also been corroborated recently^18^, where a model trained on this dataset demonstrated consistent test performance on various external COVID-19 clinical test datasets. For the second examination, known shortcuts are integrated into the COVID-positive class or COVID-negative class of the HF-train dataset using the same procedures outlined in Appendix A1.

Finally, if the trained shortcut detectives pass the two exams, i.e. AUC of close to 0.5 on the shortcut-free dataset and AUC of close to 1.0 on the shortcut-present dataset, they are referred to as certified shortcut detectives.

#### Image preprocessing and model architecture

For the MIMIC, HF, BIMCV, UW Health and MIDRC datasets, the original DICOM images are converted to 8-bit png format with a size of 224-by-224 using the default window level and window width. For the COVIDx dataset, images are directly resized to 224-by-224.

To train the shortcut detectives, five different model architectures (Table 1) that are broadly used for image classification with state-of-the-art performance on the ImageNet^19^ classification tasks are investigated in this work. Although we cannot exhaust all the possible model architectures for this purpose, the models we investigated include classic and modern Convolutional Neural Networks (CNN) and the recently introduced Swin Transformer. These models vary in architectural design and complexity (number of model parameters and floating-point operations, FLOPs). For each model architecture, an ensemble of five individually trained models with different training-validation splits are used. More technical details on the model training are shown in Appendix A2.

#### Deployment of the shortcut detectives

Certified shortcut detectives are deployed to detect shortcuts in real-world datasets, including BIMCV, UW, MIDRC, COVIDx, and RoentGen-MIMIC. We also trained two COVID-19 classification models using **HF-train** dataset and **COVIDx-train** dataset and compared their generalizability using internal and external tests.

#### Statistics

The 95% confidence intervals (CI) for the AUC were calculated using the statistical software R (version 4.0.0) with the pROC package. CIs were calculated using the bootstrap method with 2000 bootstrap replicates.

## Results

### Certification of the shortcut detectives

As shown in Table 2, the trained detectives successfully passed the two exams. Note that an AUC of 0.0 is simply due to the assignment of class labels, which is equivalent to an AUC of 1.0. Both indicate perfect classification performance. It is also shown in Table 2 that all five neural network architectures achieve a similar performance level. This result aligns well with our understanding that contrast and sharpness shortcuts are intrinsic to the dataset and the task.

### Shortcut detection in real-world datasets

Using the certified shortcut detectives, we investigated several curated COVID-CXR datasets for possible shortcuts. UW Health is a single-site, privately curated dataset; BIMCV and MIDRC are both multi-institutional public datasets; COVIDx is the first open access COVID-CXR dataset made available by the experts in the computer science community.

Similar to the detective certification process, for a COVID-CXR dataset where COVID-19-positive cases are assigned a label “1” and negative cases are given a label “0”, if the shortcut detective can differentiate the two classes (AUC significantly deviates from 0.50), it indicates the existence of a corresponding shortcut. For the RoentGen-MIMIC dataset, real CXRs are labelled as “1” and synthetic CXRs are labelled as “0”.

The results presented in Table 3 were obtained using shortcut detectives based on the DenseNet model. The results confirm the presence of image sharpness and contrast shortcuts in the COVIDx dataset, which can be exploited by models trained on such data and compromise their generalizability in real clinical settings. Conversely, the other three datasets that were curated by medical professionals exhibit no such shortcuts. We conducted a performance comparison between two models trained on COVIDx and Henry Ford datasets, respectively, which are of similar size, but the former has both sharpness and contrast shortcuts as identified by the shortcut detectives. The results displayed in Table 4 indicate that the COVIDx model exhibits poor generalization performance, as evidenced by the large AUC gap between internal and external tests. In contrast, the HF model exhibits consistent performance on both internal and external tests. Additionally, as shown in Table 3, some contrast and sharpness differences were also detected in the RoentGen-MIMIC dataset. While the generated synthetic CXRs appear visually realistic, caution must be exercised when using them for AI model development due to the potential learning of shortcuts caused by the inherent contrast and sharpness differences between real and synthetic data.

## Discussion

Shortcut learning has been a topic of interest in the machine learning community, particularly in computer vision (CV) and natural language processing (NLP). Researchers have explored shortcut learning behavior from different perspectives, such as underspecification^3^, shortcut learning in various NLP tasks^25^, and mitigation strategies for domain-knowledge agnostic models^26^. Notably, it has been observed that convolutional neural networks tend to rely on content with high spatial frequency or strong local correlations to establish connections between input and labels in CV and NLP^27,28^. However, it remains unclear whether these observations can be extended to medical diagnosis tasks, where clinical datasets have distinct characteristics from those in ImageNet. Therefore, studying shortcut learning for well-defined, clinically relevant tasks using real-world clinical datasets is crucial for medical AI applications.

In this study, we demonstrated that acquisition-dependent attributes (ADAs), such as image contrast and sharpness differences arising from the entire image generation pipeline, can serve as intrinsic shortcuts during clinical diagnosis learning tasks. Inadequate quality control procedures during data collection can allow these shortcuts to inadvertently infiltrate the curated dataset. If shortcuts contaminate the dataset, neural networks can easily exploit them during training, thereby impairing their ability to generalize to other real-world datasets.

Thus, it is imperative to identify possible shortcuts in the training dataset prior to model development. In this study, we present a methodical framework for training and validating shortcut detectives for chest X-ray classification, with emphasis on image contrast and sharpness - two essential intrinsic characteristics of chest X-ray images. However, it should be noted that these are not the only possible shortcuts that may exist in chest X-ray datasets. If other intrinsic shortcuts are suspected in a collected dataset, the general framework presented in this work can be utilized to construct similar shortcut detectives and identify alleged intrinsic shortcuts.

Once the intrinsic shortcuts are identified, it is essential to develop strategies to mitigate their impact on the learned models. One possible approach is to develop standardization and normalization techniques for image contrast and sharpness to adjust these attributes without affecting the disease features. Alternatively, examining proven intrinsic shortcut-free datasets, such as the baseline dataset (Henry Ford Health) and the three additional datasets (UW Health, BIMCV, and MIDRC) shown to be free of intrinsic shortcuts in this study, can provide further insight on how to avoid these shortcuts in the data curation process. However, it is worth noting that this is a limitation of the present work, and future research should investigate the development of mitigation strategies for the identified intrinsic shortcuts.

## Supplementary Material

Supplement 1

## Figures and Tables

**Table 1:** List of different model architectures studied in this work

Model	Number of parameters	FLOPs	ImageNet Accuracy	Year developed
VGG-16^20^	138 M	31 B	73.4%	2014
DenseNet-121^21^	8 M	5.7 B	74.4%	2017
EfficientNet^22^	54 M	24 B	85.1%	2020
Swin Transformer^23^	88 M	15 B	83.6%	2021
ConvNeXt^24^	89 M	15 B	84.1%	2022

Note: ImageNet accuracy data obtained from: https://pytorch.org/vision/stable/models.html#classification

**Table 2:** Certification of the shortcut detectives

	VGG	DENSE	Eff	Swin	Conv
**ADA(S) shortcut detective**					
Exam 1: HF-train	**0.49** [0.48,0.50]	**0.56** [0.56,0.57]	**0.56** [0.55,0.57]	**0.53** [0.52,0.53]	**0.54** [0.53,0.54]
Exam 2a: With ADA-S(+)	1.00 [1.00,1.00]	1.00 [1.00,1.00]	1.00 [1.00,1.00]	1.00 [1.00,1.00]	1.00 [1.00,1.00]
Exam 2b: With ADA-S(−)	**0.00** [0.00,0.00]	**0.00** [0.00,0.00]	**0.00** [0.00,0.00]	**0.00** [0.00,0.00]	**0.00** [0.00,0.00]
**ADA(C) shortcut detective**					
Exam 1: HF-train	**0.47** [0.46,0.48]	**0.48** [0.47,0.49]	**0.47** [0.46,0.48]	**0.49** [0.48,0.49]	**0.48** [0.47,0.48]
Exam 2a: With ADA-C(+)	1.00 [1.00,1.00]	1.00 [1.00,1.00]	1.00 [1.00,1.00]	1.00 [1.00,1.00]	1.00 [1.00,1.00]
Exam 2b: With ADA-C(−)	**0.00** [0.00,0.00]	**0.00** [0.00,0.00]	**0.00** [0.00,0.00]	**0.00** [0.00,0.00]	**0.00** [0.00,0.00]

Note: (+) means the shortcut is added to the COVID-positive CXRs; (−) means the shortcut is added to the COVID-negative CXRs.

**Table 3:** Shortcut detection on real-world COVID-CXR datasets (DenseNet model result)

	COVIDx	RoentGen-MIMIC	UW	BIMCV	MIDRC
ADA(S) shortcut	**0.84** **[0.83,0.84]**	**0.37** **[0.34,0.39]**	0.50 [0.48,0.52]	0.47 [0.45,0.48]	0.45 [0.45,0.46]
ADA(C) shortcut	**0.81** **[0.80,0.81]**	**0.05** **[0.04, 0.06]**	0.53 [0.51,0.55]	0.56 [0.55,0.57]	0.53 [0.52,0.54]

**Table 4:** Performance of two trained models

	Internal	UW	BIMCV	MIDRC
COVIDx	1.00 [1.00, 1.00]	0.60 [0.58,0.62]	0.61 [0.60,0.62]	0.57 [0.56,0.58]
HF	0.78 [0.76,0.80]	0.77 [0.76,0.79]	0.79 [0.78,0.80]	0.75 [0.74,0.75]
