# Supplementary material for: Training certified detectives to track down the intrinsic shortcuts in COVID-19 chest x-ray data sets
Source: Res Sq. 2023 Apr 28:rs.3.rs-2818347. Preprint. [Version 1] doi: 10.21203/rs.3.rs-2818347/v1 (PMC10168454; doi:10.21203/rs.3.rs-2818347/v1)
Supplement: Supplement 1 [file NIHPPRS2818347V1-supplement-1.pdf]

Supplementary Files

This is a list of supplementary files associated with this preprint. Click to download.

- Supplement.docx
